# Mercury Exposure in Mother-Children Pairs in A Seafood Eating Population: Body Burden and Related Factors

**DOI:** 10.3390/ijerph16122238

**Published:** 2019-06-25

**Authors:** Shamshad Karatela, Neil Ward, Janis Paterson

**Affiliations:** 1 Faculty of Medicine, University of Queensland, Herston QLD 4006, Australia; 2 Department of Chemistry FEPS, University of Surrey, Guildford, Surrey GU2 7XH, UK; n.ward@surrey.ac.uk; 3 School of Public Health and Psychosocial Studies, AUT University, Auckland 0627, New Zealand; Janis.paterson@aut.ac.nz

**Keywords:** hair biomarker, toxic metal, mercury, seafood, methylmercury

## Abstract

*Background:* Mercury is a neurotoxin that affects neurodevelopment in children; however, its association at the lowest concentration is not clear. The main objective of this study is to measure and evaluate mercury concentrations in mother–child pairs and its association demographics, lifestyle, and dietary factors within the Pacific Island Families living in Auckland, New Zealand. *Methods:* Mercury exposure was assessed in a sub-sample of mother–child pairs who were a part of the Pacific Island Families birth cohort, in Auckland, New Zealand at the 6-year phase. Hair samples were collected from both mothers and their children to determine mercury concentrations. Total mercury was measured using inductively coupled plasma mass spectrometry for hair samples. An interviewer-based reliable food frequency questionnaire (FFQ) examined the frequency of seafood by all the participants. Other variables such as sociodemographic (ethnicity and gender), lifestyle factors (income, education, and smoking status) and health outcomes (child behaviour and obesity) were also collected. *Results:* In this study, 41% of both mothers and their children had mercury concentrations above the US Environmental Protection Agency (EPA) recommended value of 1 µg/g. Most of the participants ate fish 3 or more times a week. A significant correlation was observed between mother and child hair mercury concentrations (Spearman Rho 0.79 (95% confidence interval (CI): 0.65, 0.88)). *Conclusions:* Mercury levels in children can be affected by their mothers’ levels due to similar eating patterns.

## 1. Introduction

Exposure to toxic metals in seafood-eating communities, especially women of childbearing age and children, is a serious health concern that requires further attention, especially low-level chronic toxic metal inputs via the diet or environmental pathways [1]. Toxic metals and their associated compounds, such as methylmercury (MeHg), can act as neurodevelopmental toxicants that cause damage to the developing brain and nervous systems [2]. Although there is a complex interplay between genetics, biological, and environmental factors in the aetiology of neurodevelopmental disorders, cognition and abnormal metabolism [3], continuous, low-level exposures to Hg, especially MeHg concentrations, can exacerbate the accumulation in the body causing adverse effects, especially during gestation and in children [4]. Additionally, children tend to absorb metals at a greater rate than adults due to their smaller body size [5]

Pacific people are known to consume a high-fish diet in New Zealand (NZ) [6]. An NZ survey found that over half (53%) of Pacific people are more likely to eat fresh or frozen fish/seafood one or more times a week than those categorised as NZ European and Others (NZEO) [6]. Seafood-eating communities are at a greater risk of mercury or methylmercury exposures [1,2] as consuming predatory fish can expose people to Hg or MeHg [7]. Predatory fish, such as marlin, tuna, and shark, can bioaccumulate Hg or MeHg [8]. According to the WHO (2014) [9], the subsistence fishing populations cognitive impacts were observed in children that consumed fish containing mercury (i.e., between 1.5/1000 and 17/1000). Additionally, a meta-analysis described the relationship between methylmercury and intelligent quotient (IQ) and showed a linear relationship between 1 µg/g increase in maternal hair mercury concentrations and 0.18-point decrease in IQ [10]. The exposure data that are a representative of all populations is scarce which makes the global burden of disease difficult to estimate. However, an estimation has reported that the incidence rate for mild mental retardation is estimated to be as high as 17.37 per 1000 infants born among a subsistence fishing population in the Amazon, resulting in a loss of 202.8 disability-adjusted life years per 1000 infants [11].

There is limited knowledge of low-level postnatal mercury exposure using hair as a biomarker within the Pacific Island population who have migrated to NZ from the Pacific Islands. The main objective of this study was to measure and evaluate low-level postnatal hair mercury exposures in mother–child pairs using inductively coupled plasma mass spectrometry (ICP-MS) instrumentation to determine mercury concentrations in NZ. Therefore, this study aimed to explore the distribution of mercury through seafood diet in Pacific children and their mothers and to identify any factors that may be associated with mercury concentrations using scalp hair as a biomarker for Hg exposure.

## 2. Materials and Methods

### 2.1. Study Design and Population

This descriptive study was conducted between June 2007 and June 2008 within the Pacific Island Families (PIFs) cohort when the children were six years of age and living in South Auckland. The PIF study is a birth cohort that has been following over a thousand Pacific children and their families [12,13]. A sub-sample of six-year-old children and their mothers were recruited from the PIF study (total: *n* = 92 (mother–child pairs: *n* = 46)). Maternal interviews and hair sample collection were conducted in the home setting to those who had consented to participate. Children were excluded if participants (both children and their mothers) had very short hair. This study was approved by the NZ Health and Disability Ethics Committee (NTX/07/05/050).

### 2.2. Measurement of Variables

#### 2.2.1. Exposure Measure: Total Mercury Concentrations

Scalp hair samples were used for determining mercury exposure as it is the recommended biomarker for estimating the methylmercury dose received by the child’s brain [14]. Hair closest to the scalp represents the latest mercury exposure (approximately one month’s growth) [15,16]. As elements, such as mercury, occur in scalp hair at higher levels, it allows for more sensitive and more analytically accurate results [17].

The vast majority of systemic exposure to organic mercury is exposure to the methyl species. However, the methylmercury is converted into inorganic mercury before it is bound to hair [17,18], and so total mercury in hair samples was measured for this research. Additionally, there is a strong correlation between long-term systemic exposure to organic mercury and its hair concentrations [17] and therefore scalp hair samples provided a direct measurement of mercury burden in children and mothers in this sample. Hair mercury concentrations were measured as a continuous variable in µg/g.

The US Environmental Protection Agency (EPA) has recommended a concentration of 1 µg/g [19] and hence this criterion was used for this research.

#### 2.2.2. Variables

The demographic variables included in this study were ethnicity (Samoan, Tongan, and Others), gender (male and female), and household income levels (categorised into <$20,000, $20,000–$40,000, >$40,000), smoking status (yes and no), and children’s body size (normal, overweight, and obese) using the International Obesity Task Force (IOTF) criteria, and maternal age (categorised into 20–29, 30–39, and 40–49 years) was also collected. This study utilised the parental version of the 120-item Child Behaviour Check List (CBCL)/6–18 from maternal PIF study participants regarding the behaviour of their six-year-old children and was administered by the PIF interviewers. Children that scored within the clinical range were classified as having behavior problems and those that scored lower were classified as normal.

Mothers were asked during the interviews to identify themselves and their children’s ethnicity and, in the case of multiple ethnicities, to specify the main ethnicity (Samoan, Tongan, Cook Island, Niuean, Other Pacific, and Non-Pacific). The seafoods most eaten within this population in NZ were identified by visiting local fish shops in South Auckland (where most of the participants resided), all local fish sold were included in a questionnaire, and local names were provided. All participants had to tick the most eaten fish in their household. Food frequency questions asked were based on the past week and as to how often they had eaten fish and chips, fish, salted fish, and raw fish and were recoded as binary variables separately (<3 times per week; >3 times per week) for this study due to small numbers. Mothers were also asked about fishing habits (yes and no). The average weight and height of children were calculated according to the procedures documented in an operation manual from which the IOTF [20] criteria were derived from. Prior to data collection, equipment was standardised before weight and height data were collected.

### 2.3. Data Collection Procedures

Scalp hair samples were chosen and collected as a biomarker since these were less invasive (especially in children) than blood or urine samples and therefore would be easily collected. Once consent/assent was obtained, questionnaires were administered and hair samples were collected at that same visit if possible. Other appointments were made for those participants who could not answer the questionnaires at the time or who provided hair samples during the first visit.

#### Hair Sample Collection, Storage, and Handling

Scalp hair samples were collected as a biomarker for determining mercury. The procedure for hair sample collection was adapted from the World Health Organisation [14] method. These hair samples were cut from the back of the head close to the scalp with stainless steel scissors which was cleaned with a steriliser solution after every cut for hygiene reasons. Approximately 50–100 µg (~50 strands) of scalp hair were collected from all participants (mother–child pair). The part of hair closest to the scalp was tied with a cotton thread so that it could be identified for sample analysis. The samples were then placed in a polythene bag closed with a zipper (all samples were given code numbers so that participants were not directly identifiable). These were then stored in a locked cabinet at room temperature until chemical analysis.

### 2.4. Hair Sample Laboratory Analysis

In recent years, ICP-MS has enabled human tissues, such as scalp hair, to be accurately analysed for mercury and many other toxic metals. Additionally, hair is not entirely influenced by metabolic processes and regulatory mechanisms of the body unlike blood which does not replicate the actual concentrations [21].

The scalp hair samples were sub-sampled in a clean glovebox, especially for cases of very long hair. A 2 cm length of the hair (referenced against the scalp end) or approximately 0.5–1.0 g dry weight of hair was then washed with neutral detergent (1:100) in 20 mL glass vials and rinsed well with distilled water. The sample was then washed with acetone and dried under reduced pressure. The samples were then cut into fine pieces in the vial using a pair of dissection scissors. They were stored in a desiccator until analysis. ICP-MS measurements were undertaken using an Agilent 7700× ICP-MS spectrometer (Agilent Technologies, Santa Clara, CA, USA). Multi-element standards of mercury (201 Hg isotope with a 13.18% abundance) and other elements under investigation were prepared by appropriate dilution from stock standards (BDH, Aristar^®^, Mumbai, India) in 1% (*v*/*v*) HNO_3_ acid (Fisher Scientific, Trace Analysis Grade). A trace amount of gold salt was added to HNO_3_ to preserve all forms of mercury. The gold ion acts as a strong oxidizing agent that converts or maintains mercury as mercuric ion which remains in solutions. Thus, a solution of 2% (*v*/*v*) HNO_3_ containing 200 µg/L Au was used for preparation of all samples and standards. The routine determination of Hg by ICP-MS was affected by a pronounced memory effect in the sample introduction system. This resulted in long washout times for the analyte, which affected the accuracy and reliability of the analytical procedure. To minimise the memory effect, the system was washed using 2% (*v*/*v*) HNO_3_ containing 200 µg/L Au between samples. Eight working calibration standards were prepared to cover the linear dynamic range of 1–500 µg/L. All calibration curves were plotted as a concentration against a ratio of the analyte counts per second (CPS) signal (blank corrected) and internal standard CPS signal (for mercury 209Bi+) and had R-squared values of at least 0.999. The elemental values for each hair sample were corrected for the dilution factor resulting in the final values for statistical analysis. A NIES No. 13 Human Hair Certified Reference Material (CRM) (National Institute for Environmental Studies, Tsukuba City, Japan) was used to validate the method used. Twelve replicates (using the same mass and volume as the scalp hair samples) were analysed and gave a mean of 4.32 (standard deviation: ±0.38) µg/g Hg dry weight value compared against the mean certified value of 4.42 (standard deviation: ±0.20 µg/g) Hg dry weight.

### 2.5. Statistical Analysis

All data obtained for this study were stored in Microsoft Excel databases (Microsoft Corporation, Redmond, WA, USA). The data were cleaned; range and consistency checks were performed and coded for analysis. Questions with no response were not included in the analysis as a distinct coded variable. The cleaned data were then exported to STATA version 10 [22] for statistical analysis.

Normality was tested by visually inspecting normal distribution plots and histograms as well as residual plots of dependent variables. Summary statistics for non-normally distributed mercury in mothers and children were calculated to include median, upper (75%), and lower (25%) quartiles concentrations. Spearman correlation was computed to understand the concordance between mothers and children. Non-normally distributed maternal and child Hg concentrations were tested using the Kruskal–Wallis test for more than two categories and the Mann–Whitney test for comparing two categorical variables. *p* < 0.05 (95% confidence interval (CI)) was considered as the cut-off value for statistical significance.

## 3. Results

### Demographic Characteristics and Mercury Distribution

More than half of all the participants (mother and children) were of Samoan ethnicity, (63%) followed by Tongan (30%), and the remaining (7%) were from the Others (European, Māori, and Niuean) group within this sample. Their average annual household income was between $21,000 and $39,000 at the six-year phase. Out of the 46 children, there were more girls (57%) than boys (44%). Most of the participants ate fish 3 or more times a week (78%). About 35% of the children were either overweight or obese (using the IOTF criteria [18]. With regards to behaviour problems (using the CBCL criteria), there were only 15% with behaviour problems in the clinical range as reported by mothers. Mullet was the most eaten fish (41%) followed by salmon/tuna (24%). Almost half of the mothers smoked cigarettes.

Mothers had slightly higher median concentrations of hair mercury (median: 0.43 µg/g) than their children (median: 0.32 µg/g). A significant correlation was observed between mother and child hair mercury concentrations (Spearman Rho 0.79 (95% CI: 0.65, 0.88). There were some mothers (61%) and their children (22%) with hair mercury concentrations above 1 µg/g with the highest level being 2.5 µg/g for mothers and 1.67 µg/g for children (Table 1).

There were significant differences in the children’s mercury levels and IOTF obesity criteria, frequency of fish eaten, maternal smoking, and family income (Table 2). Within the mother’s group, significant differences were found for mercury concentrations and maternal age category, annual household income, fishing, and frequency of fish eaten (Table 3).

## 4. Discussion

This research used scalp hair samples to explore the relationship between low-level hair mercury concentrations in children and their mothers whose staple diet was fish within the PIF birth cohort at the 6-year phase. The sociodemographic (gender, income levels, maternal age, and ethnicity), dietary factors (seafood consumption and type of fish eaten), as well as a life-style factor (maternal smoking), and health outcomes (IOTF obesity and child behavior) in relation to hair mercury concentrations in both mothers and children were explored.

Total hair mercury concentrations in participants (children and their mothers): Around 41% of both mothers and their children had hair mercury concentrations higher than the US EPA’s recommended concentrations of 1 µg/g (17). The highest range of hair mercury concentration in mothers was 2.5 µg/g and in their children it was 1.67 µg/g. Mothers had slightly higher median mercury concentrations than children (0.32 µg/g vs 0. 43 µg/g) and this is because adults are bigger and eat bigger portion sizes than children. A recent study in NZ, also within the PIF study, found that 21% of 9-year-old children had toenail mercury concentrations of between 1.5 µg/g and 6 µg/g [23]. Another NZ study conducted in the late seventies, where maternal hair samples were collected on 1000 mothers consuming three fish meals a week, resident in the North Island, had an average hair mercury level above 6 µg/g [24]. That hair mercury level was much higher than the median hair mercury concentration being 0.43 µg/g Hg in mothers in the current study. However, these participants ate shark tissue, a predatory fish (higher in Hg), but shark is no longer eaten as much in NZ as reported in an NZ review [2]. The participants in the current study did not report eating shark.

### 4.1. Seafood Diet and Mercury Exposure

The majority of the participants (mothers and children) ate fish more than 3 times per week (78%). The most eaten fish was mullet in this study (mother: 59%; children: 41%), followed by snapper and tuna/salmon (children: 34%; mothers: 20%). Slightly higher mercury concentrations were observed in mothers that ate mullet, although the difference between fish types and hair mercury was not significant. Mullet fish are generally safer to eat as they are known to have lower mercury concentrations [25] but it seems that these mullet-eating participants had slightly higher mercury concentrations than those that ate other kinds of fish within the mothers. Other food items may also contain higher mercury concentrations, so fish may not be the only exposure factor. For example, the 2009 NZ Total Dietary Survey found that some adults consumed certain kinds of teas that had higher mercury concentrations [26]. Perhaps, future research should also consider other food items that may expose participants to mercury.

Catching fish was a predictor of mercury concentrations in mothers within this study. Generally, fishing is very common amongst PIFs [27] but fish caught cannot be monitored for mercury exposure or other toxic metals like imported or exported fish [28]. Predatory fish that feed at deeper depths in the open ocean, like swordfish and marlin, have higher mercury concentrations [29] than those that feed in waters near the surface, like yellowfin tuna and snapper. Fishing in the deep sea ocean may be a concern; however, in this research, the question on where they fished or type of fish caught was not asked.

### 4.2. Demographic Factors (Ethnicity and Gender)

There are only a few gender-related susceptibility studies on mercury exposure and the results that are available are inconclusive [2,30]. Amongst the few, two studies have found that male children are affected more by neurotoxins than females [31,32]. The effect of gender on mercury concentrations was not significant in these children. Further research on this aspect is required to establish the effect of gender on susceptibility to mercury exposure.

Ethnicity was not a predictor of mercury exposure within this study in both mother and child groups. This is contrary to other studies that have shown that metabolic excretion rates might vary according to ethnicity [33,34,35]. For example, Vietnamese and Chinese experience a genetic polymorphism for enzymes involved in ethanol metabolic excretion and Hg export from cells [35,36] which could affect elimination rates. Animal studies have found this evidence as well [37]. Differences in MeHg concentrations have been found among children from different ethnic groups within a population not considered at risk of MeHg exposure [38]. Thus, different sub-groups may have different metabolic processes of MeHg. Additionally, traditional food consumption and/or cooking habits may decrease the metabolic absorption or increase the excretion of MeHg. However, this theory has yet to be proven [34,39]. There is also some evidence that selenite (from selenium) has a protective effect on inorganic Hg levels [40,41], but there is no evidence regarding protection against MeHg [34]. The concentration of selenium in the NZ population is generally lower than in many other countries [42]. Selenium and its effects on mercury have been demonstrated in other research [43,44]. The selenium concentration was inversely associated with mercury concentration in a recent Pacific Island study on 9-year-old children [23]. However, more research is required on this aspect within Pacific people. Other studies have concluded that the beneficial influence of nutrients (such as selenium and omega-3 fatty acids) from fish may counter any adverse effects of MeHg on the developing nervous system [45]. Again, the results are equivocal. For example, a Faroe Islands birth cohort study investigated omega-3 fatty acids and selenium as potential modifiers of the effects of MeHg exposure through fish/seafood diet but found no such associations [46]. Accordingly, Choi and Grandjean (2008) [47] suggested in their review that, to assess the full impact on the toxicity of MeHg and the beneficial effects of nutrients, both the good and bad effects should be assessed at the same time in order to separate opposite impacts on the outcomes. Cooking methods can also have an effect on Hg bioavailability; for example, fish that is boiled or fried has lower Hg concentrations than raw fish [48]. Therefore, monitoring and providing proper advice on mercury exposures for mothers-to-be and children is highly recommended.

### 4.3. Health Outcomes (Smoking, Child Behavior, and Obesity)

In children, an effect was observed on mercury concentrations in relation to maternal smoking. Almost half of the mothers smoked and this activity exposes their children to passive smoking. Tobacco products contain numerous toxicants, such as toxic metals [49], as tobacco plants extract many of the toxic chemicals from the soil and accumulate in the lamina. Smoking is a major cause of exposure to heavy metals in the blood and organs of smokers [50,51]. These can then be transferred to smokers and passive smokers via cigarette smoking. Some of the mercury exposures in this sample in both mothers and children could be from smoking and passive smoking though type of cigarettes was not determined. Further research is required in passive smoking exposure within the Pacific Island people in NZ.

In this research, there was no association between behavior problems (as determined by the maternal reported child behavior checklist) in the children and scalp hair mercury concentrations. Most of the international studies have measured developmental disorders in children with pre- and postnatal mercury exposures [49]. However, the results have been mixed [2,52,53].

Scalp hair mercury concentrations in children showed an association on the IOTF obesity criteria. Similarly, a Korean National Health and Nutrition Examination Survey IV, showed that higher body mass index (BMI) was a predictor of higher blood mercury levels [54]. These findings were in contrast to another study that did not find an association with BMI and mercury exposure [55]. With the discrepancies between published studies, more well-designed research is required to understand childhood obesity in relation to mercury exposures. Mothers BMI was not measured in the study; however, futures studies should include maternal BMI as well.

### 4.4. Relationship Between Mother and Child Mercury Concentrations

With respect to mother–child pairs, there was a strong correlation (*r* = 0.79) in hair mercury concentration between the mother and child which shows that there is a common source of exposure in these mother–child pairs. Diet seems to be the most likely course of mercury exposure as the levels in scalp hair reflect primarily exposure to MeHg from food sources [56]. However, smoking and passive smoking could be another pathway for mercury exposure in this population of mother–child pairs. These results are similar to another study of mother–child pairs with a correlation of Hg levels between children and their mothers (*r* = 0.72) [57]. Other studies have also shown a correlation in mother–child pairs on hair mercury concentrations similar to that in this current study [58,59].

### 4.5. Strengths and Limitations

The study limitations and strengths need to be considered while interpreting the results. The major strength for this research was that it was carried out within a robust PIFs birth cohort which is a representative of the PIFs living in NZ. As this was a descriptive study, it does not differentiate between cause and effect nor the direction of the events [60]. Other study designs to be considered for future research include case-control studies which are quicker, less expensive, and less burdensome on participants as well. Although the best study designs are experimental studies, this may not be always feasible due to financial, time, and ethical restrictions. The statistical inference was limited by a small sample (*n* = 92 mother–child pairs). Thus, it is likely that there was insufficient power to detect any health effects. Specifically, due to the small sample size, there is a risk of Type II errors, which means that it fails to reject a null hypothesis [61]. Large sample sizes usually produce narrow CIs and therefore more precise results [61]. Apart from the small sample size leading to very low statistical power, the strength of this study was that it has provided an insight into the collection of scalp hair samples within the Pacific Island people in NZ. We measured total mercury which included both methylmercury and inorganic mercury [62,63,64]. The study controlled for various confounding factors; however, there could be some residual confounding due to unknown or unmeasured factors.

The questionnaires used in this research were reliable and validated but they have some limitations. Inaccuracies of measurements (misinterpretation of questions) across the sample could have contributed to a non-differential measurement error in exposure and outcome which can sometimes lead to a bias towards finding no effect [65]. A limitation of the food frequency questionnaire is that mothers were required to recall their usual weekly consumption of foods which is subject to bias rather than measuring the actual intakes [66,67]. Mothers were proxy for children’s eating habits which may lead to over- or under-estimating their frequency of food intakes. Other research has shown under-reporting of energy intake by young Pacific adults and in parental reports of intake of their children [68].

## 5. Conclusions

The main determinates of hair mercury concentrations in both children and their mothers were the frequency of eating fish and annual household income. Additionally, fishing determined mercury concentrations in mothers and maternal smoking influenced mercury concentrations in children. About 41% of the participants had mercury concentrations slightly higher than the US EPA’s recommended concentrations. There was no association between low-level mercury concentrations and behaviour problems but an association was found with regards to children’s obesity and mercury concentrations. This study has enhanced the understanding of low-level hair mercury concentrations in mothers and their children of PIFs living in South Auckland, NZ and the factors that may influence mercury concentrations.

## Figures and Tables

**Table 1:** Mothers and children with mercury levels below and above 1 µg/g Hg.

Variables	⩽1 µg/g Hg *n* (%)	>1 µg/g Hg *n* (%)
Children	36 (78)	10 (22)
Mothers	18 (39)	28 (61)

**Table 2:** The median, 25th, and 75th percentiles of total mercury concentrations (µg/g Hg) in Pacific Island children’s hair samples.

Variables	*n* (%)	Median (25th, 75th)	*p*-Value ^a^
Children	46	0.32 (0.2, 0.6)	
Gender			0.3 ^
Girls	26 (57)	0.3 (0.2, 0.5)	
Boys	20 (43)	0.3(0.2, 0.4)	
IOTF obesity			0.04 *
Normal	14 (30.4)	0.3 (0.3, 0.6)	
Overweight	18 (39.1)	0.4 (0.2, 0.7)	
Obese	14 (30.4)	0.6 (0.5, 0.8)	
Child behavior			0.7 ^
Yes	7 (15)	0.4 (0.2, 0.6)	
No	39 (85)	0.3 (0.2, 0.7)	
*Ethnicity*			0.3 *
Samoan	29 (63)	0.3 (0.2, 0.5)	
Tongan	14 (30)	0.3 (0.1, 1.2)	
Others	3 (7)	1.03 (0.2, 2)	
Type of fish eaten			0.5 *
Snapper	11 (23.9)	0.4 (0.2, 1.02)	
Mullet	19 (41.3)	0.3 (0.2, 0.7)	
Salmon/Tuna	16 (34.9)	0.3 (0.2, 0.5)	
Frequency of fish eaten			**<0.05 ^**
<3 times per week	6 (13)	0.4 (0.2, 1.3)	
>3 times per week	40 (87)	0.3 (0.03, 1.7)	
Fish and chips			0.23 ^
<3 times per week	21 (46)	0.4(0.2, 0.7)	
>3 times per week	25 (54)	0.3 (0.2, 0.6)	
Fish cakes and sticks			0.24 ^
<3 times per week	33 (71.7)	0.3 (0.2, 0.5)	
>3 times per week	13 (28.3)	0.3 (0.2, 1.1)	
Fish raw			0.3 ^
<3 times per week	25 (54.4)	0.2 (0.2, 0.6)	
>3 times per week	21 (45.7)	0.3 (0.2, 0.8)	
Maternal smoking			**0.04 ^**
Yes	22 (48)	0.2 (0.1, 0.5)	
No	24 (52)	0.6 (0.2, 0.5)	
Annual household income			0.04 *
<$20,000	12 (26)	0.2 (0.2, 1.2)	
$20,000 to $40,000	19 (41)	0.4 (0.2, 0.5)	
>$40,000	8 (17.4)	0.7 (0.4, 0.8)	

^a^ The values were calculated using the Kruskal–Wallis test or Mann–Whitney test. ^ represents the Mann–Whitney test and * represents the Kruskal–Wallis test. *p* < 0.05 was considered as significant and is highlighted in bold. *n* was the number of participants. IOTF: International Obesity Task Force.

**Table 3:** Pacific Island mothers hair mercury concentrations (µg/g Hg, dry weight) based on their demographics.

Variables	*n* (%)	Median (25th, 75th)	*p*-Value ^a^
Mothers	46	0.43 (0.21, 0.8)	
Age category			0.04 *
20 to 29	7 (15)	0.4 (0.3, 1.4)	
30 to 39	30 (65)	0.4 (0.2, 1.6)	
40 to 49	9 (20)	0.6 (0.3, 1.8	
Ethnicity			0.7 *
Samoan	29 (63)	0.5 (0.2, 1.6)	
Tongan	14 (30)	0.4 (0.3, 1.2)	
Others	3 (7)	0.4 (0.3, 1.2)	
Annual household income			0.6 *
<$20,000	12 (26)	1.0 (0.2, 1.8)	
$20,001–$40,000	19 (41)	0.5 (0.2, 1.4)	
>$40,000	8 (17)	0.4 (0.3, 1.2)	
Maternal smoking status			0.5 ^
Smoking	22 (48)	0.5 (0.2, 1.8)	
Non-smoking	24 (52)	0.4 (0.2, 1.5)	
Fishing			**0.03 ^**
Yes	18 (39)	0.5 (0.3, 1.6)	
No	28 (61)	0.34 (0.2, 1.6)	
Type of fish eaten			0.3 *
Tuna/Salmon	9 (20)	0.2 (0.2, 1.3)	
Snapper	10 (22)	0.3 (0.4, 1.6)	
Mullet	27 (59)	0.5 (0.3, 1.7)	
Frequency of fish eaten			**0.02 ^**
<3 times per week	14 (30)	0.3 (0.2, 1.7)	
>3 times per	32 (70)	0.6 (0.4, 1.6)	
Fish and chips			0.2 ^
<1 times per week	16 (17)	0.4 (0.2, 0.9)	
>3 times per week	30 (33)	0.7 (0.3, 2)	
Fish cakes and sticks			0.4 ^
<3 times per week	34 (37)	0.5 (0.2, 1.6)	
>3 times per week	12 (13)	0.3 (0.2, 1.3)	
Fish raw			0.4 ^
<3 times per week	24 (26)	0.5 (0.2, 1.4)	
>3 times per week	22 (24)	0.5 (0.3, 1.6)	

^a^ The values were calculated using the Kruskal–Wallis and Mann–Whitney test. ^ represents the Mann–Whitney test and * represents the Kruskal–Wallis test. *p* < 0.05 was considered as significant and is highlighted in bold. *n* was number of participants.
